# Development and performance evaluation of a high‐speed multileaf collimator

**DOI:** 10.1002/acm2.12026

**Published:** 2016-12-29

**Authors:** Xiang Zhang, Peiqing Ye, Hui Zhang

**Affiliations:** ^1^ Department of Mechanical Engineering Tsinghua University Beijing 100084 China; ^2^ Beijing Key Lab of Precision/Ultra‐Precision Manufacturing Equipments and Control Tsinghua University Beijing 100084 China; ^3^ The State Key Laboratory of Tribology Tsinghua University Beijing 100084 China

**Keywords:** dosimetric properties, HS‐MLC, mechanical properties, performance evaluation

## Abstract

Multileaf collimator (MLC) tracking represents a promising technique for motion management in radiotherapy. However, the conflict between limited leaf speed/acceleration and the demand for tracking fast target motion is now a prominent issue. Conventional MLCs typically have a maximum leaf speed of 3–4 cm/s and a maximum leaf acceleration of 50–70 cm/s^2^, which are inadequate to track fast target motion. To cope with this problem, we have recently developed a high‐speed multileaf collimator (HS‐MLC) prototype, which employs linear motors instead of rotary motors to drive leaves. Consequently, it inherits various benefits of linear motors, including direct drive and high dynamics. The primary aim of this paper was to introduce the development and performance evaluation of the HS‐MLC. The evaluation includes Monte Carlo simulations of the basic dosimetric properties, camera‐based measurements of the mechanical properties and tracking experiments for 25 sets of patient‐measured motion data. The Monte Carlo simulation results show that the maximum leakage at 6MV is 1.29% and the average is 0.61%. The end‐to‐end leakage is 3.96% for 5 cm offset and is 1.75% for 10 cm offset. The penumbra for a standard 10 × 10 cm^2^ field ranges from 4.8 mm to 5.4 mm across the full range of leaf motion. The mechanical property measurements demonstrate that the maximum leaf speed is 40 cm/s, the maximum leaf acceleration is 1000 cm/s^2^, and the geometric accuracy can be kept within 0.5 mm. Regarding the tracking experiments for a wide range of motion patterns (fast breathing, irregular breathing, etc.), a root‐mean‐square error (RMSE) of less than 0.19 mm was achieved. In conclusion, the HS‐MLC is able to well track fast target motion that is beyond the capability of conventional MLCs due to its superior mechanical properties. The new MLC design provides a feasible solution to make high‐accuracy and high‐efficiency motion management possible.

## 1 Introduction

In radiotherapy, intrafractional motion induced by respiration may cause large discrepancy between delivered and planned dose distributions.^1^ Several techniques accounting for intrafractional motion have been proposed, including motion encompassing, respiratory gating, multileaf collimator (MLC) tracking, etc.^2^ Among them, MLC tracking is realized through dynamically adapting the aperture to the moving target, which does not rely on margin enlargement or beam hold‐offs (unless large position error occurs) in contrast to the first two. Previous studies have applied MLC tracking to dealing with different forms of target motion: one‐dimensional (1D) translation,^3^, ^4^, ^5^, ^6^ two‐dimensional (2D) translation,^7^, ^8^, ^9^ three‐dimensional (3D) translation,^10^ rotational motion^11^ and deformation.^12^

Plenty of studies have pointed out that limited leaf speed/acceleration is a major constraint to tracking capability.^13^, ^14^, ^15^, ^16^ To keep up with a moving target, the maximum leaf speed should at least exceed the maximum target speed in the case of target motion parallel to leaf direction. Moreover, the requirement for maximum leaf speed is much higher in the case of target motion perpendicular to leaf direction.^13^, ^17^ Conventional MLCs typically have a maximum leaf speed of 3–4 cm/s,^18^ whereas the target motion is likely to exceed this speed and even can reach up to 9.4 cm/s.^19^ Therefore, for conventional MLCs, tracking fast target motion is beyond their capability, especially when the perpendicular component is large. Beam hold‐offs occur when the position error exceeds the machine tolerance, which inevitably sacrifices efficiency.

To remove or reduce violation of the MLC mechanical constraint—namely the occurrence of beam hold‐offs, several software‐based efforts have been made to date, such as optimal leaf sequencing algorithm^4^, ^7^, ^14^ and moving average algorithm.^15^ Nevertheless, there still remain some limitations using these algorithms. An alternative solution is to optimize structure of MLC to achieve higher leaf speed/acceleration. For conventional MLCs, the leaf speed/acceleration is limited mainly because the rotary motors adopted cannot provide adequate torque. One way to increase the torque is to adopt new rotary motors with larger power. But this is probably not allowed because larger power rotary motors require larger installation space. An alternative way is to replace rotary motors with other variants. The binary MLC employs air cylinders to drive leaves, achieving an extremely fast speed of around 2.5 m/s (slice thickness, 5 cm; switching time, 20 ms).^20^ However, the binary MLC has only two states—wide open and fully closed because it was first designed for tomotherapy only.

To simultaneously achieve the two goals, that i, high leaf speed/acceleration and continuous leaf trace control, we have recently developed a novel high‐speed multileaf collimator (HS‐MLC) prototype, which employs linear motors as the drive elements. Linear motors are especially well known for the advantages of direct drive and high dynamics, which provide the possibility to increase leaf speed/acceleration considerably. This paper mainly aims to introduce the development and performance evaluation of the standalone HS‐MLC which is a non‐integrated non‐radiated newly developed MLC system. First, the description of the HS‐MLC and position measurement system is presented. Then, the assessments of the basic dosimetric properties through the Monte Carlo simulations and of the mechanical properties through the camera‐based measurement system are presented. Afterward, the tracking experiments for 25 sets of patient‐measured respiration data are reported. The final section summarizes this paper and looks into the future work.

## 2 Methods

### 2.A Description of the HS‐MLC and position measurement system

#### 2.A.1 Mechanism design of the HS‐MLC

**Figure 1:**
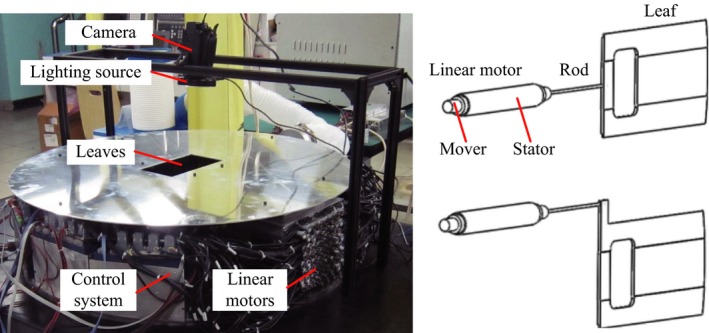
Left: Picture of the HS‐MLC prototype as well as the camera‐based measurement system. Right: schematic drawing of the linear motor driven leaf and two connection types are shown.

Figure 1 shows the HS‐MLC prototype as well as the camera‐based measurement system (discussed in details in the section that follow). The HS‐MLC consists of 128 leaves, 8 cm in height, 0.625 cm in projected width and 6 cm in overtravel at isocenter, which define a maximum field size of 20 × 40 cm^2^. The key geometric features of the leaves include rounded leaf end, divergent leaf sides and tongue and groove side profile. The material is tungsten alloy with a density of 18 g/cm^3^ (95% tungsten). Further details of the HS‐MLC are listed in Table 1. Each leaf is driven by a linear motor, which consists of a stator (made of coils) and a mover (made of magnets). The electro‐magnetic interaction between the stator and the mover produces axial thrust force directly. The mover is mechanically connected to either the body of the leaf or the extended portion of the leaf through a rigid bar (see the right side of Fig. 1). No transmission mechanisms such as gear‐rack or screw‐nut are needed to convert rotation to linear motion. The leaf position is detected through a position sensor mounted inside the linear motor.

**Table 1:** Physical dimensions of the HS‐MLC

Item	Value
Number of leaves	128
Leaf height	8 cm
Leaf width at isocenter	0.625 cm
Maximum overtravel at isocenter	6 cm
Maximum field at isocenter	20 × 40 cm^2^
Radius of leaf end	29.6 cm
Air gap	0.014 cm
Source to collimator distance (SCD)	38 cm
Source to axis distance (SAD)	100 cm
Leaf density	18 g/cm^3^

#### 2.A.2 Control framework of the HS‐MLC

**Figure 2:**
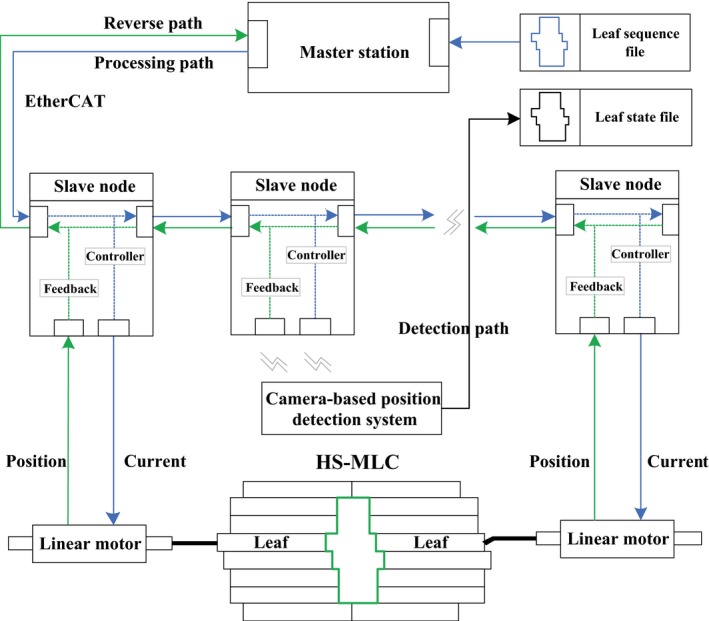
Framework of the control system.

The framework of the control system is shown in Fig. 2. It mainly consists of a master station (embedded PC) and 32 slave nodes (microprocessors). Each slave node controls 4 linear motors based on the controller. The EtherCAT bus is adopted as the bridge between the master station and the slave nodes benefiting from its high transmission rate (maximum transmission rate, 100 Mbit/s) and high‐accuracy clock synchronization (less than 1 *μ*s). In addition, the three data flow paths (processing path, reverse path, and detection path) can be clearly seen from Fig. 2. The command trajectories stored in the leaf sequence file are transmitted from the master station to the slave nodes through the processing path (blue) while the feedback of the 128 actual leaves trajectories sampled by the inside position sensor are returned to the master station through the reverse path (green). Afterward, through the detection path (black), the actual position obtained by the camera‐based position detection system is stored in the leaf state file.

#### 2.A.3 Camera‐based position detection method

**Figure 3:**
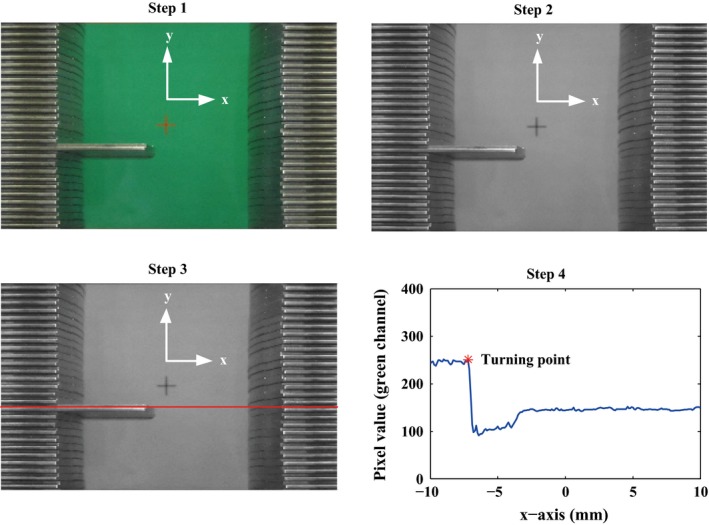
Steps of imaging processing. The bottom panel compares the outputs of the camera and the inbuilt position sensor.

A camera‐based measurement system (Sony HDR‐CX520) was constructed to evaluate the mechanical properties (see Fig. 1). This measurement technique was first adopted by Keall et al.^21^ and Sawant et al.^10^ to evaluate the geometric accuracy of a DMLC tracking system. To facilitate the subsequent image processing, a green plate with a cross‐mark was placed under the aperture (see Fig. 3). The frame rate of the camera is 25 Hz, which means the interval between two image frames is 40 ms. Each image frame contains 1920 × 1080 pixels. Adjust the camera's field of view to 9.6 × 5.4 cm^2^, which corresponds to a resolution of 0.05 mm. As the geometric dimension of the cross‐mark is known in advance, it is used as a standard to calibrate the leaf position in case that the camera's field of view is not exact. A Matlab program was developed for the image processing, as illustrated in Fig. 3. Step 1: extract a RGB image from the video. Step 2: convert it to a grayscale image (extract the green channel of the RGB image). Step 3: locate the central line of the leaf. Step 4: plot the “pixel value versus *x* coordinate” curve.

**Figure 4:**
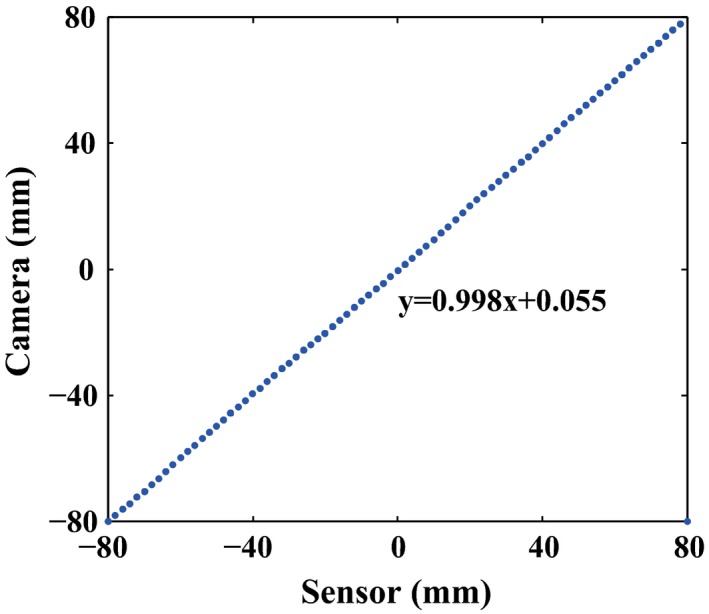
Comparison between the camera and inbuilt sensor.

Considering that the colors of the leaf and the plate contrast against each other greatly, the curve changes abruptly at the intersection between the leaf and the plate. However, due to the impact of the rounded leaf end and the shadow, it is difficult to determine which point on the curve is exactly the leaf tip. We use the first turning points of adjacent image frames to calculate the relative leaf position, rather than the absolute leaf position. Through this way, the systematic error is eliminated. For further validation, the comparison between the camera and the inbuilt position sensor was made, as shown in Fig. 4. They show good consistency with a high correlation coefficient.

### 2.B Evaluation of the dosimetric properties

For the HS‐MLC design above, we apply the Monte Carlo simulations for evaluation of the basic dosimetric properties. Monte Carlo simulations are widely accepted as an accurate numerical method for dose calculation.^22^, ^23^, ^24^ The Monte Carlo codes EGSnrc/BEAMnrc were used.^25^ To model the HS‐MLC, the VARMLC component module (CM) was adopted because they have similar geometric features. A mono energetic source of 6 MeV and a Gaussian intensity distribution with a full width at half maximum (FWHM) of 0.12 cm were selected. The electron cutoff energy (ECUT) was set to 0.7 MeV and the photon cutoff energy (PCUT) was set to 0.01 MeV. The phase space file was scored at a plane below the HS‐MLC (50 cm from the target) with a total of 109 histories. Then, the dose calculations at a depth of 1.5 cm and source‐to‐surface distance (SSD) of 98.5 cm in the water phantom were performed by importing the phase space file into DOSXYZnrc code. All the dose values were normalized to the output of a 10 × 10 cm^2^ field. The size of the water phantom utilized in DOSXYZnrc is 20 × 20 × 20 cm^3^ while the phantom is divided into several voxels whose size is 0.05 cm perpendicular to the leaf motion direction, 0.5 cm in direction of the leaf motions, and 0.5 cm high. Only when the relative variance of dose of each voxel reaches 1% can the simulation be terminated.

### 2.C Evaluation of the mechanical properties

The evaluation of the mechanical properties involves the maximum leaf speed and maximum leaf acceleration, geometry accuracy and the capability of the HS‐MLC in tracking target motion. For each measurement, a leaf sequence file was generated and then transmitted to the master station. Once the file was executed, the actual trajectories of the selected leaves were extracted from the camera‐based measurement and stored in the leaf state file. Discrepancies between the command and actual trajectories can be obtained through analyzing the two files and transformed to their projections at the isocenter plane. The control cycle was set to 1 ms in these measurements, which would introduce little latency into the system.

#### 2.C.1 Maximum leaf speed and maximum leaf acceleration

**Figure 5:**
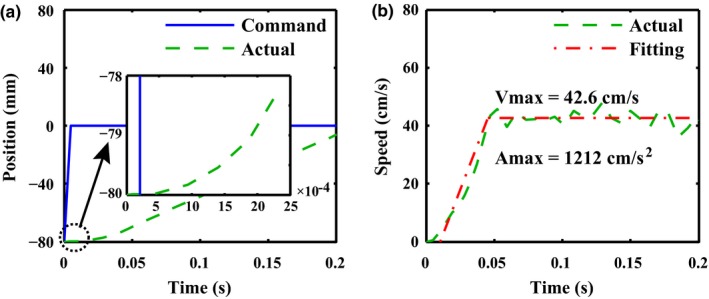
Maximum leaf speed and maximum leaf acceleration measurement. The left panel compares the command trajectory and the actual trajectory. The right panel plots the actual speed and the corresponding fitting curve. *A*_*max*_ is equal to the slope of the first segment of the fitting curve and *V*_*max*_ is equal to the amplitude of the second segment of the fitting curve.

In order to determine the maximum leaf velocity, and acceleration, a leaf sequence file based on a ‘step‐shaped’ command trajectory was created (see Fig. 5(a)). Such a request was far beyond the capability of the HS‐MLC. In the absence of acceleration and maximum leaf‐velocity constraints, as a consequence, the leaf would first speed up to the maximum speed *V*_max_ at full acceleration *A*_max_ and then maintain this speed. Through this way, both *V*_max_ and *A*_max_ could be determined. As the acceleration period was very short, the inbuilt position sensor was used for this measurement instead of the camera on account of the finite frame rate of the camera.

#### 2.C.2 Geometric accuracy

**Figure 6:**
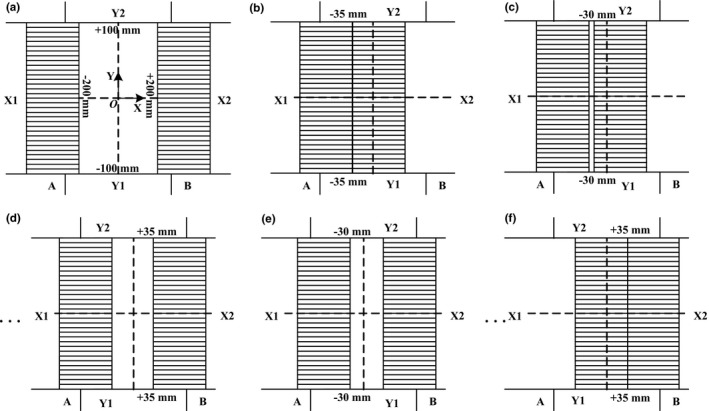
Panel (a) and panel (b) illustrates the MLC leaves in motion for measurement of static error. In panel (a), MLC leaves start from the closed position and 32 B (right) leaves are requested to move and stop alternately to +3.5 cm with an interval of 0.5 cm. In panel (b), MLC leaves start from the opening position and 32 (a) (left) leaves motion is from −3.5 cm to +3.5 cm with an interval of 0.5 cm. In the end, whole MLC leaves stop at the position of +3.5 cm and the position error is recorded when the leaf stopped and stayed stable at each step.

**Figure 7:**
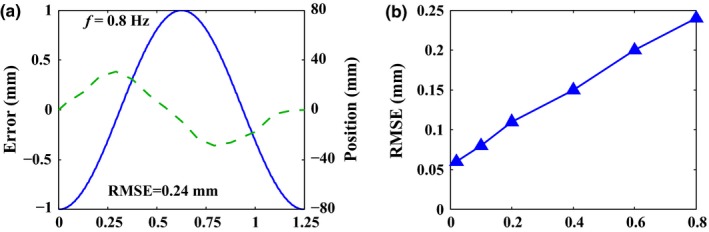
Tracking accuracy measurement under different sinusoidal command trajectories. (a) The tracking error as quantified by the difference between command trace and actual leaf trajectory is plotted in the frame of Fig. 9(a) (green line) when the frequency of the command trace is 0.8 Hz. (b) the variation tendency of the RMSE is exhibited for each frequency of command trace.

The geometric accuracy includes two aspects: static accuracy and tracking accuracy. The static accuracy refers to the position accuracy when the leaf stops at a certain position, which may vary with the position and the motion direction of the leaf. A typical leaves motion pattern in Fig. 6 was created. 32 A (left) leaves and 32 B (right) leaves would be initially positioned at −3.5 cm and requested to move and stop alternately to +3.5 cm with an interval of 0.5 cm. Figure 6 shows the leaves of the right bank B moving first, followed by the motion of the left bank A. Then, the position error was recorded when the leaf stopped and stayed stable at each step. The tracking accuracy, on the other hand, refers to the position accuracy when the leaf follows a dynamic trajectory. A series of sinusoidal command trajectories were created. The amplitude of each trajectory was fixed to 8 cm and the frequency *f* was 0.02 Hz, 0.1 Hz, 0.2 Hz, 0.4 Hz, 0.6 Hz and 0.8 Hz, respectively. The corresponding peak leaf speed occurred at *t* = 1/(4*f*) and *t* = 3/(4*f*) was around 1 cm/s, 5 cm/s, 10 cm/s, 20 cm/s, 30 cm/s and 40 cm/s, respectively, which covered a wide speed range. Figure 7(a) shows the typical sinusoidal command trajectory with the frequency of 0.8 Hz. Under this circumstance, the position error was recorded per control cycle.

#### 2.C.3 Leaf–leaf variations

For evaluation of leaf–leaf variations, 32 adjacent leaves of right bank B were selected to synchronously track the same sinusoidal trajectory shown in Fig. 7(a). According to the actual trajectories extracted from the camera‐based system, we can then demonstrate whether every measured leaf in each snapshot has good consistency with the motion trajectories in the leaf sequence file.

### 2.D Evaluating the capability of the HS‐MLC in tracking target motion

**Figure 8:**
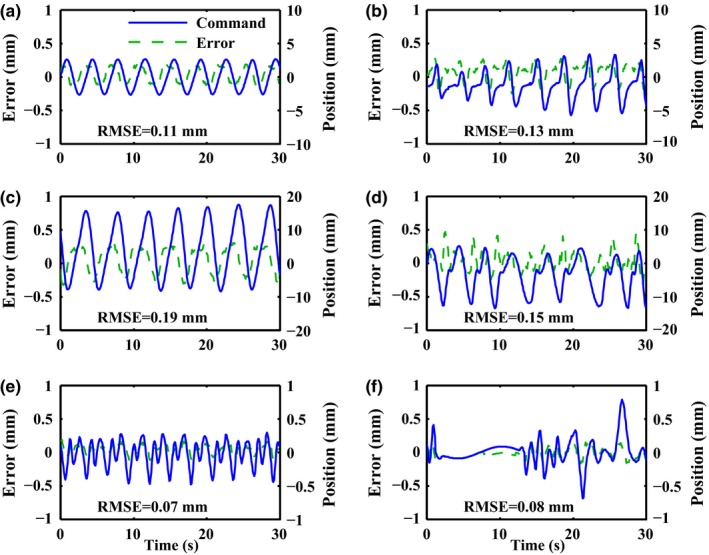
Experimental results of tracking 6 sets of patient‐measured motion trajectories. The blue line in each figure represents the patient‐measured motion trajectories while the green dash line indicates the tracking error. The corresponding RSME is also illustrated in each panel.

A total of 25 sets of patient‐measured motion data (provided by Namkung Kim, University of Ulsan, Korea) were selected for the tracking experiments. Figure 8 displays some representatives covering various motion patterns: (a) regular breathing; (b) varying amplitude; (c) large amplitude; (d) varying frequency; (e) high frequency; (f) transient excursion. Assume the leaf direction is parallel to the motion direction in this study. These motion data were sampled with a rate of 30 Hz. To match this value, the control cycle of the HS‐MLC system was also set to 30 Hz. Each tracking experiment lasted for 30 s, during which the position error was recorded per control cycle.

## 3 Results

### 3.A Dosimetric properties

**Figure 9:**
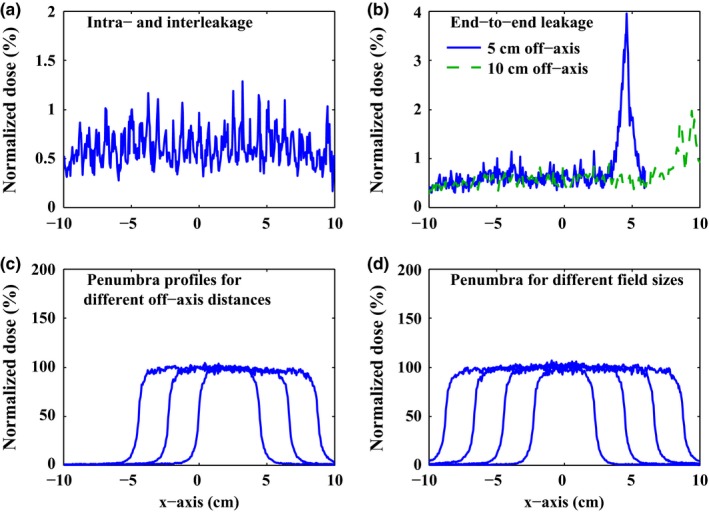
Monte Carlo simulation results for the HS‐MLC. (a) Intra‐ and inter leakage. (b) end‐to‐end leakage with 5 cm and 10 cm off‐axis distance. (c). penumbra profiles for different off‐axis distances (2.5 cm, 5 cm, 7.5 cm for a 5 × 5 cm^2^ field size). (d) penumbra for different field sizes (5 × 5 cm^2^, 10× 10 cm^2^, 15 × 15 cm^2^, 20 × 20 cm^2^).

As seen in Fig. 9(a), the maximum leakage through the leaves is 1.29% and the average is 0.61%, compared to 0.63% and 0.37% of 160 MLCTM (Siemens AG, Erlangen, Germany) and compared to 1.8% and 1.5% of Varian Millennium MLC‐120 (Varian Medical Systems, Inc., Palo Alto).^26^ The results meets IEC (International Electrotechnical Commission)^27^ requirement which allows a maximum of 2% and an average of 0.75%. The end‐to‐end leakage is 3.96% at 5 cm off‐axis distance and is 1.75% at 10 cm offset, as seen in Fig. 9(b). For a standard 10 × 10 cm^2^ field, the 80%/20% penumbra ranges from 4.8 mm to 5.4 mm across the full range of leaf motion, as seen in Fig. 9(c). The penumbra profiles for different field sizes (5 × 5 cm^2^, 10 × 10 cm^2^, 15 × 15 cm^2^, 20 × 20 cm^2^) are displayed in Fig. 9(d), showing that the 80%/20% penumbra varies from 5.0 mm to 5.9 mm.

### 3.B Mechanical properties

#### 3.B.1 Maximum leaf speed and maximum leaf acceleration

Figure 5 plots the results of tracking a ‘step‐shaped’ command trajectory. As expected, the leaf could not keep up with the command trajectory and hence a large tracking error appeared. This forced the leaf to move at the maximum acceleration *A*_max_ until the maximum speed *V*_max_ was reached. As shown in Fig. 5(a), after the command signal given to the HS‐MLC, it maintains stationary in several control cycles and does not respond to the command signal simultaneously. However, the mechanical latency is less than 5 ms based on the local enlarged drawing. In consideration of the fluctuation of the actual speed (see green dash line in Fig. 5(b)), a fitting curve composed of two segments was constructed (see red dash dot line in Fig. 5(b)). Accordingly, *A*_max_ was equal to the slope of the first segment, that is, 1212 cm/s^2^, and *V*_max_ to the amplitude of the second segment, that is,. 42.6 cm/s. Their nominal values were determined to be 1000 cm/s^2^ and 40 cm/s, respectively.

#### 3.B.2 Geometric accuracy

**Figure 10:**
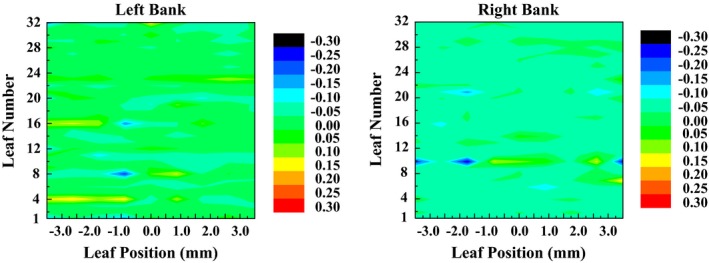
Static accuracy measurement. The static position error of both 32 leaves of left bank A and 32 leaves of right bank B at each step are illustrated.

Figure 10 plots the results of the static accuracy measurement. Based on the static position error of both 32 leaves in left bank A and 32 leaves in right bank B at each step, it was observed that the maximum error lied within 0.25 mm, which was specified as the static accuracy. In addition, it can be seen that the static position error varies with leaf number and leaf position because of the existence of various mechanical connections and assembly structures.

Figure 7 displays the results of the tracking accuracy measurement. The tracking error which is quantified by the difference between command trace and actual leaf trajectory is plotted in the frame of Fig. 7(a) (green line). The maximum position error occurred when the leaf speed reached the peak at *t* = 1/(4*f*) or *t* = 3/(4*f*)) basically. When the frequency reached 0.8 Hz, the corresponding peak leaf speed was around 40 cm/s. This represented the most challenging scenario because the peak leaf speed reached the maximum leaf speed *V*_max_. In this scenario, a maximum position error of around 0.4 mm was observed, which was identified as the tracking accuracy. Furthermore, as shown in Fig. 7(b), the variation tendency of the root‐mean‐square error (RMSE) is exhibited for different frequencies. Obviously, with the increase in the frequency, the RMSE tended to be larger.

#### 3.B.3 Leaf–leaf variations

**Figure 11:**
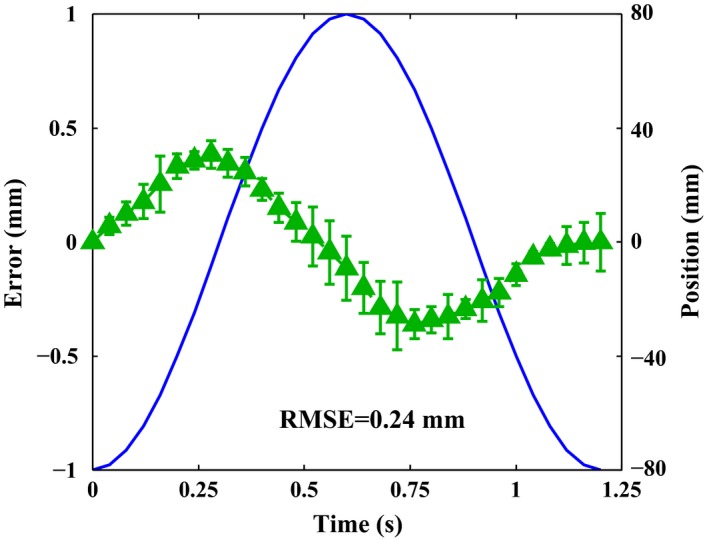
**(**a) comparison of the actual trajectories of 32 adjacent leaves of right bank (b) when following the same sinusoidal trajectory where the green triangle represents the average leaf deviation while the green short line denotes the standard deviation.

A comparison of the actual trajectories of 32 adjacent leaves of right bank B when following the same 0.8 Hz sinusoidal trajectory is shown in Fig. 11. The average leaf deviation (see green triangle in Fig. 11) is defined as the average tracking error of various leaves at each sampled leaf position while the standard deviation (see green triangle in Fig. 11) refers to the maximum difference between the average leaf deviation and tracking error of various leaves at its corresponding sampled leaf position. The leaf–leaf variation accuracy is within 0.3 mm which has demonstrated that slight inconsistency in manufacturing and assembly exists between various leaves.

#### 3.C Performance of tracking target motion

Figure 8 displays the results of tracking six sets of representative patient‐measured motion trajectories (the remaining 19 are not displayed). For all the cases, a RMSE of less than 0.19 mm was achieved and the maximum position error could be kept within 0.5 mm, that is, the tracking accuracy. The position error tended to be larger when the peak‐to‐peak amplitude of the motion was higher, that is, the corresponding leaf speed was higher. This trend was consistent with that when tracking sinusoidal trajectories. In contrast, the irregularity of the motion trajectories seemed to have less influence (see Figs.8(e) and 8(f)). Particularly, as shown in Figs. 8(b) and 8(f), the HS‐MLC has the potential to track the sudden and small alternation of the breathing motion. In all, sub −0.5 mm tracking accuracy was achieved for the 25 sets of motion trajectories, showing robustness of the HS‐MLC in handling a wide range of motion patterns.

## 4 Discussions and conclusions

We have for the first time introduced a high‐speed multileaf collimator (HS‐MLC). The major innovation is the application of linear motors instead of conventional rotary motors to drive leaves, which provides new insight into MLC design. Performance evaluation of the HS‐MLC is done and the main results are summarized as follows:

1. Benefiting from high dynamics of linear motors, the HS‐MLC achieves a maximum leaf speed of 40 cm/s and a maximum leaf acceleration of 1000 cm/s^2^. The two parameters have been improved considerably compared with those of conventional MLCs. As a consequence, the HS‐MLC is able to well track fast target motion that is beyond the capability of conventional MLCs. This is guaranteed by two factors. On one hand, rigid rods are used to connect the linear motors and the leaves rather than gear‐rack or screw‐nut mechanisms such that the mechanical backlash is eliminated thoroughly. On the other hand, the EtherCAT bus is adopted as the interface between the master station and the slave nodes, which allows a short control cycle and introduces little latency into the system.
2. The Monte Carlo simulation was utilized to evaluate the basic dosimetric properties of the HS‐MLC. The HS‐MLC is constructed with a mono energetic source of 6 MeV, and in combination with a FWHM of 0.12 cm. Then, the numerical calculation can be achieved with the maximum leakage of 1.29% and average leakage of 0.61%. The magnitude of the observed results is acceptable for the IEC specification requirements. Moreover, the 80%/20% penumbra for different field sizes (5 × 5 cm^2^, 10 × 10 cm^2^, 15 × 15 cm^2^, 20 × 20 cm^2^) varies from 5.0 mm to 5.9 mm. It is essential to keep in mind that different boundary conditions in simulations have an influence on numerical results and impede direct comparability. Within this limitation, the dosimetric properties generated by simulation appear to be similar to the range of other MLCs used in clinical applications.
3. The geometric accuracy has been promoted greatly compared to earlier MLC designs with the rotary motors as the driving components. The HS‐MLC achieved a tracking accuracy of sub −0.5 mm over the full speed range from 0 to 400 mm/s while the maximum static error lied within 0.35 mm. The tracking accuracy exhibits a clear dependence of position error on leaf speed as the variation tendency shown in Fig. 9(b). The higher is leaf speed, the larger is position error. This is attributed to the fact that higher speed causes higher friction of the guide and raises higher demand for the control system. Moreover, taking the inconsistency in mechanical connections, assembly structures and manufacturing into consideration, the leaf–leaf variations measurements for the 32 adjacent leaves of right bank B are conducted. The result showed that the variations did not exceed 0.3 mm, which is reasonable and essential for efficient MLC tracking.
4. To assess the tracking performance, 25 sets of patient‐measured respiratory motion trajectories are conducted on the HS‐MLC. For all the tracking experiments, the HS‐MLC achieved the RMSE of less than 0.2 mm and maximum position error less than 0.5 mm, that is, the tracking accuracy, proving robustness of the HS‐MLC in handling a wide range of motion patterns. It should be noted that the regular breathing trajectory in Fig.11(a) is approximated to a sinusoidal trajectory with frequency of 0.25 Hz. We compared the RMSE with that in Fig. 9(b): the former had lower leaf speed but the same level of tracking error existed. This result violated the aforementioned trend because the control cycle was increased from 1 ms to 33.3 ms here to match the sampling rate. Thus, the control points were less and as a result the control performance was decreased.

Further development and promotion of the HS‐MLC is still in progress and several issues remain to be addressed.

**Figure 12:**
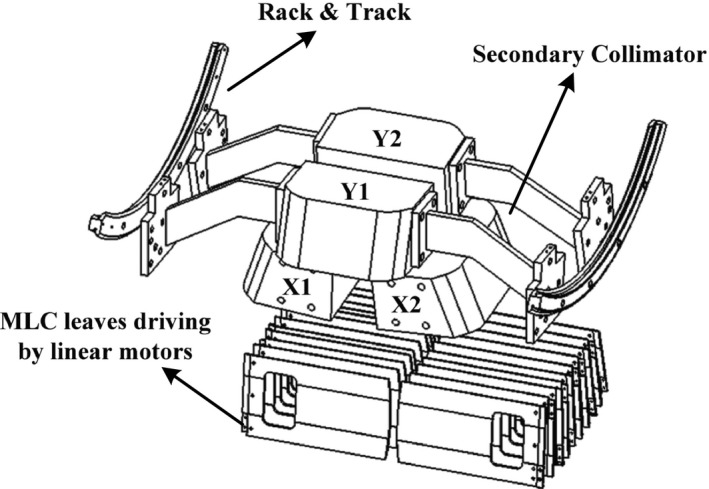
Schematic description of MLC position in the head.

1. With some hardware and software modifications, there is still a great potential to further promote performance of the HS‐MLC. The linear motors can provide higher thrust force by increasing their current, but at the expense of producing more heat. This needs a stronger cooling system to avoid overheating of the linear motors. The geometric accuracy, on the other hand, can be increased through the following aspects. First, since the geometric accuracy is mainly subjected to the friction of the guide, better coating material and better lubricant are required. Second, by combining the adopted proportion‐integration‐differentiation (PID) control algorithm with other advanced algorithms, better control performance can be achieved. In addition, for discrete motion data with a low sampling rate, it is necessary to insert more control points between two sampling points to improve the control performance.
2. The HS‐MLC was evaluated as an independent system in this paper. It is available to be integrated into an accelerator to evaluate its overall performance since the prototype HS‐MLC was within clinical requirements according to the IEC regarding MLC on a linac. Actually, to examine the mechanical and dosimetric performance, the prototype MLC can be mounted on a self‐developed linear accelerator whose source to axis distance (SAD) and source to collimator distance (SCD) are 1000 mm and 380 mm, respectively. Based on the schematic of the collimator head in the linac, as illustrated in Fig. 12, the collimator head involved two groups of adjustable opposing tungsten alloy blocks (X1, X2, Y1, and Y2 in Fig. 12). With the aim of reducing the interleaf leakage and leaf transmission in IMRT treatments, both of them are located above the MLC leaves. Furthermore, each individual block implements the same mechanism of rack and track system for movement and can be separately driven by an electric motor.

Considering incorporation with the collimator head, the influence of the sag in linac secondary collimator and MLC carriage on delivery quality should be explored. To implement tracking tasks in practice, it also should be incorporated with imaging system and it is necessary to investigate whether the image acquiring accuracy and the latencies from other subsystems may impair the tracking performance.^28^, ^29^

- Only target motion parallel to the leaf motion direction is considered in this paper. Under this assumption, the HS‐MLC can achieve 100% efficiency due to the fact that the achieved maximum leaf speed (40 cm/s) far exceeds the maximum target motion speed (9.4 cm/s, reported by Shirato et al. 2006).^19^ However, the other forms of target motion also widely exist. It has been reported that large perpendicular translational motion^10^ and large rotational motion^11^ are likely to cause very low efficiency. Therefore, it is worth studying whether the HS‐MLC is capable to compensate for other forms of target motion without loss of efficiency.
- When the HS‐MLC is used for intensity modulated radiotherapy therapy (IMRT) treatments, how to maximize its performance is a crucial problem. The leaf motion in IMRT serves two functions: one for delivering the IMRT plan, the other for synchronizing with the target motion. This raises a question about how to allocate the maximum leaf speed between the two functions. Allocating more to the former contributes to reducing monitor units, treatment time and given radiation dose to organs at risk;^30^, ^31^ allocating more to the latter allows tracking faster target motion. Therefore, the allocation strategy needs to be well optimized to balance the two functions.

In conclusion, with superior mechanical properties, the HS‐MLC is highly promising for high‐accuracy and high‐efficiency motion management in the future.
